# Single-Institutional Experience of Chronic Intracranial Electroencephalography Based on the Combined Usage of Subdural and Depth Electrodes

**DOI:** 10.3390/brainsci11030307

**Published:** 2021-02-28

**Authors:** Yutaro Takayama, Naoki Ikegaya, Keiya Iijima, Yuiko Kimura, Suguru Yokosako, Norihiro Muraoka, Kenzo Kosugi, Yuu Kaneko, Tetsuya Yamamoto, Masaki Iwasaki

**Affiliations:** 1 Department of Neurosurgery, National Center Hospital, National Center of Neurology and Psychiatry, Kodaira, Tokyo 187-8551, Japan; ytakayama@ncnp.go.jp (Y.T.); iijimakeiya@ncnp.go.jp (K.I.); yuikkmr@ncnp.go.jp (Y.K.); yksksgr@gmail.com (S.Y.); norihiromura@gmail.com (N.M.); kensan03977@ncnp.go.jp (K.K.); y-kaneko@ncnp.go.jp (Y.K.); 2 Department of Neurosurgery, Yokohama City University Graduate School of Medicine, Yokohama 236-0004, Japan; nikegaya@ncnp.go.jp (N.I.); y_neuros@yokohama-cu.ac.jp (T.Y.)

**Keywords:** epilepsy, electrode implantation, subdural electrode, depth electrode, electrocorticogram, presurgical evaluation

## Abstract

Implantation of subdural electrodes on the brain surface is still widely performed as one of the “gold standard methods” for the presurgical evaluation of epilepsy. Stereotactic insertion of depth electrodes to the brain can be added to detect brain activities in deep-seated lesions to which surface electrodes are insensitive. This study tried to clarify the efficacy and limitations of combined implantation of subdural and depth electrodes in intractable epilepsy patients. Fifty-three patients with drug-resistant epilepsy underwent combined implantation of subdural and depth electrodes for long-term intracranial electroencephalography (iEEG) before epilepsy surgery. The detectability of early ictal iEEG change (EIIC) were compared between the subdural and depth electrodes. We also examined clinical factors including resection of MRI lesion and EIIC with seizure freedom. Detectability of EIIC showed no significant difference between subdural and depth electrodes. However, the additional depth electrode was useful for detecting EIIC from apparently deep locations, such as the insula and mesial temporal structures, but not in detecting EIIC in patients with ulegyria (glial scar). Total removal of MRI lesion was associated with seizure freedom. Depth electrodes should be carefully used after consideration of the suspected etiology to avoid injudicious usage.

## 1. Introduction

Chronic intracranial electroencephalography (iEEG) is performed to investigate patients with drug-resistant epilepsy in whom the epileptogenic zone is not clearly identified by non-invasive presurgical evaluations. The indications and approaches for iEEG are changing based on the recent introduction of less invasive therapeutic devices and robot-assisted stereotactic implantation systems for stereo-electroencephalography (SEEG) [1,2,3]. SEEG is now considered the gold standard in most of the patients due to low morbidity [4]. However, implantation of subdural electrodes through craniotomy is still widely performed in Japan, partly because of the limited availability of these new devices. Stereotactic implantation of depth electrodes is frequently combined with subdural electrodes to explore deep-seated activities. Subdural electrodes, especially subdural grids, have been reported to have a slightly higher complication rate than depth electrodes [5]. However, fatal hemorrhagic complications due to depth electrode insertion were rarely reported in SEEG cases [6], and the use of depth electrodes requires careful consideration. The primary aim of this study is to clarify the efficacy and limitations of the combined implantation of subdural and depth electrodes under craniotomy for presurgical evaluation of epilepsy. To achieve this purpose, we first compared the detectability of iEEG seizure onset patterns between subdural and depth electrodes and then investigated the relationship of our approach, the combined implantation, to seizure outcome.

## 2. Materials and Methods

Clinical information, including characteristics of the patients and their epilepsy, locations of epileptogenic zone, details of electrode implantation, postoperative seizure outcomes, and etiologies of epilepsy, was collected retrospectively from our database. The study was approved by the ethics committee at the National Center of Neurology and Psychiatry, Tokyo, Japan (No. A2017-028).

### 2.1. Subjects

This study included 53 patients (28 females) with drug-resistant epilepsy who underwent combined implantation of subdural and depth electrodes for chronic iEEG before resective epilepsy surgery in our institution, with at least one year postoperative follow up. A total of 72 pediatric and adult patients with intractable epilepsy underwent chronic iEEG from May 2016 until December 2019. We combined subdural and depth electrodes in principle, trying to explore surface and deep-seated cortex simultaneously. Ten patients who were evaluated only with depth electrodes or with subdural electrodes were excluded from the study. Three patients were evaluated only with depth electrodes because subdural electrodes were not placed due to previous craniotomy. Seven patients were evaluated with subdural electrodes alone because they had no relevant deep-seated area to be explored. Two patients did not proceed to resective surgery, and seven patients were excluded for inadequate follow-up periods. Clinical characteristics of the patients are summarized in Table 1. 

Mean age at electrode implantation was 17.6 years (range, 3 to 53 years). Mean duration of follow up was 27.5 months (range, 12.2 to 49.5 months). The most common seizure symptom was impaired awareness seizures including behavioral arrest (45.3%).

### 2.2. Presurgical Comprehensive Epilepsy Evaluations

All 53 patients underwent presurgical comprehensive epilepsy evaluations, including medical interview, neurological and neuropsychological examinations, long-term video-electroencephalography, magnetic resonance imaging (MRI), ^18^F-fluorodeoxyglucose-positron emission tomography (FDG-PET), and magnetoencephalography. Epilepsy histories were obtained from the patient and relatives, and the semiology of their habitual seizures was confirmed. Video-electroencephalography was recorded with the standard 10–20 system of electrode placement, including bilateral anterior temporal electrodes. Three-tesla brain MRI was performed, including three-dimensional fluid-attenuated inversion recovery, double inversion recovery, and T1- and T2-weighted imaging. Epilepsy diagnosis and classification were established after the comprehensive evaluations. The location of the epileptogenic zone was estimated, and the indication of iEEG monitoring was determined at a patient-management conference. 

### 2.3. Electrode Implantation

Electrode implantation was performed under general anesthesia, intended to localize the epileptogenic zone and to define the extent of resection. Our concept of the combined implantation of depth electrodes was different from SEEG but was rather an extension of the subdural electrode implantation with open craniotomy. Craniotomy was designed to expose the suspected epileptogenic cortices for subdural electrode implantation. Depth electrodes were implanted first using stereotactic devices such as a neuronavigation system (VarioGuide^®^; Brainlab AG, Munich, Germany) or a Leksell stereotactic frame (Elekta AB, Stockholm, Sweden). Subsequently, the dura mater was widely opened, and strip or grid subdural electrodes were placed under direct vision.

### 2.4. iEEG Monitoring

**Figure 1:**
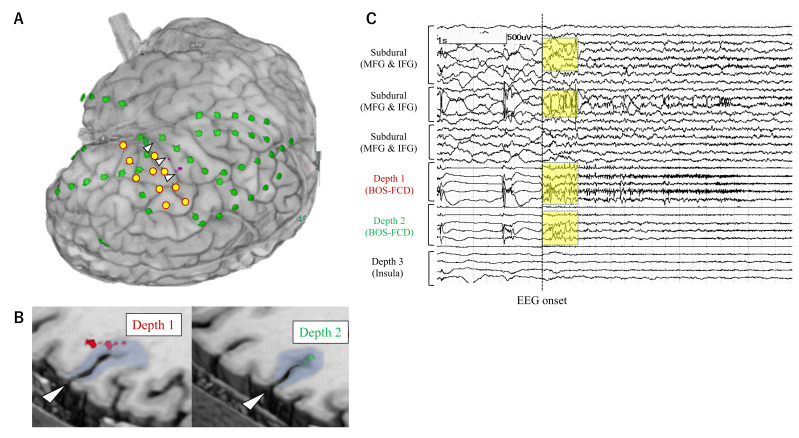
Electrode placement in a representative patient (Case 26) with the bottom-of-sulcus type FCD. (**A**) Subdural electrodes (grids and strips) (green) were placed on the surface of the right frontal, parietal, and temporal lobes, including the cortex around the abnormal sulcus (white arrowhead). Depth electrodes (pink) were additionally inserted from the right inferior frontal gyrus. The yellow circles show the subdural contacts that detected EIIC. (**B**) Depth electrodes were targeted to the abnormal cortices at the sulcal bottom of the right frontal lobe (Depth 1 and 2) and to the insular cortex (Depth 3). (**C**) On the intracranial EEG, the burst of polyspikes (yellow squares) antecedent to low-voltage fast activity was defined as EIIC, which was detected by both subdural and depth electrodes. Based on the intracranial EEG results, the area of EIIC was completely resected in addition to the FCD lesion. The patient did not have any seizures postoperatively, except for only one seizure when the medication was partly withdrawn. Abbreviations: BOS = bottom-of-sulcus, FCD = focal cortical dysplasia, IFG = inferior frontal gyrus, MFG = middle frontal gyrus.

**Figure 2:**
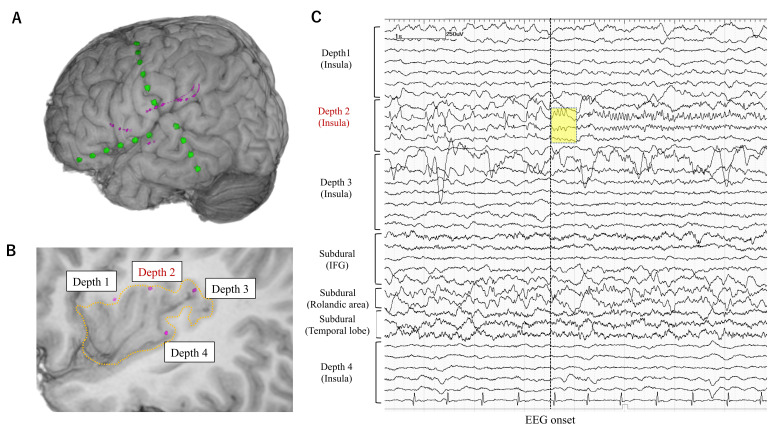
Electrode placement in a representative patient (Case 2) in whom depth electrodes were stereotactically implanted into the insular cortex. The patient had an increased T2 signal lesion suggestive of focal cortical dysplasia in the left insular cortex colocalized with glucose-hypometabolism. His ictal symptoms were characterized by the painful sensation and tonic contraction of the right hand and/or face. The ictal EEG showed rhythmic theta activity in the left fronto-temporo-central region, but no epileptiform discharges were seen interictally. (**A**) Subdural electrodes were placed for the purpose of exploring the seizure onset zone as well as for functional mapping. One of the subdural strip electrodes (green) was placed on the Rolandic area considering the seizure semiology, and the other two were placed for frontal and temporal language mapping. (**B**) All depth electrodes (pink) were targeted to the left insular cortex (surrounded by dashed line). (**C**) On the intracranial EEG, the EIIC (yellow square) was detected by only depth electrodes in the insular cortex. Depth electrodes were useful in this case in detecting the earliest ictal activity. Abbreviations: IFG = inferior frontal gyrus.

**Figure 3:**
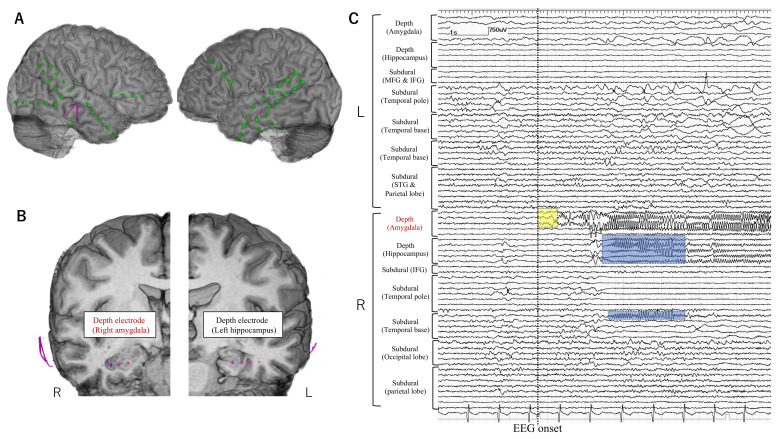
Electrode placement in a representative patient (Case 5) in whom depth electrodes were stereotactically implanted into the bilateral mesial temporal structures. The patient’s ictal symptoms were characterized by abdominal aura followed by impaired awareness. MRI showed bilateral mesial temporal hyperintensity prominently in the left side, colocalized with glucose-hypometabolism. Epileptiform discharges were localized in the right temporal region both in the ictal and interictal EEG as well as in the interictal MEG. (**A**,**B**) Subdural electrodes (green) were placed mainly on the bilateral temporal cortices. Depth electrodes (pink) were additionally inserted into the bilateral mesial temporal structures. (**C**) On the intracranial EEG, EIIC (yellow square) was detected only by depth electrodes in the right amygdala and propagated to the hippocampus and the mesio-basal temporal cortex (blue square). Depth electrodes were useful in this case in detecting the earliest ictal activity. Abbreviations: IFG = inferior frontal gyrus, MFG = middle fontal gyrus, STG = superior temporal gyrus.

iEEG monitoring was started from the day of electrode implantation. The sampling rate for the EEG recording was set at 1000 or 2000 Hz. The mean duration of iEEG monitoring was 9 days (3 to 22 days). Habitual seizures were recorded in all patients. Seizures with the EEG onset preceding the clinical onset were selected for further analysis. EEG onset for each seizure was determined as the timing at which unequivocal EEG changes appeared with the onset of seizure. Ictal EEG changes that started without >500 msec temporal delay from the EEG onset were defined as early ictal iEEG change (EIIC) (Figure 1, Figure 2 and Figure 3). Ictal iEEG changes that started clearly later than the timing of EEG onset were not defined as EIIC. Detection of EIIC by subdural electrodes, by depth electrodes, or by both was determined. The cortical area under the electrodes showing EIIC was interpreted as the seizure onset zone, which was an important indicator for determining the extent of resection. The iEEG findings were reviewed at an EEG conference attended by board-certified epileptologists. The morphological patterns of EIIC were classified into seven patterns defined in the previous report by Perucca et al. [7]. Functional cortical mapping with direct cortical stimulation was performed if necessary.

### 2.5. Resective Surgery

Indication of resective surgery was determined by board-certified neurosurgeons and epileptologists based on both non-invasive and invasive evaluations. The extent of resection was determined not only by the anatomical location of the lesion but also by the electrophysiological findings based on iEEG even in the patients with a focal MRI lesion. The cortex beneath the subdural or depth electrode contacts showing EIIC was resected unless it overlapped with the functionally important area. The planned resection was also guided by abnormal MRI and/or ^18^F-fluorodeoxyglucose-positron emission tomography (FDG-PET) areas, especially in patients with poor iEEG localization. 

### 2.6. Surgical Outcome

Postoperatively, the patients were followed up through outpatient visits or admissions for evaluation. Postsurgical seizure outcome was assessed using the International League Against Epilepsy (ILAE) seizure outcome classification [8]. 

### 2.7. Etiology

The etiology of epilepsy was determined by the histopathological diagnosis, the radiological findings, and the clinical history. 

### 2.8. Factors Related to the Detectability of EIIC

The detectability and morphological patterns of EIIC were compared between the subdural and depth electrodes, and the association of the detectability of EIIC and low-voltage fast activity (LVFA) was also investigated with the clinical factors including etiology, location of the epileptogenic zone, extent of MRI lesion, concordance between FDG-PET abnormality and MRI lesion, and seizure outcome. Fisher’s exact test was used to determine the association between categorical variables. A *p*-value of less than 0.05 was considered statistically significant.

### 2.9. Factors Related to the Seizure Freedom

In order to know the impact of the removal of EIIC on postoperative seizure outcome, we assessed whether the MRI lesion was completely resected and whether the area of EIIC was completely resected by comparing pre- and postoperative MRIs. Multiple logistic regression analysis was performed to predict postoperative seizure freedom. Complete removal of MRI lesion, complete removal of EIIC, and temporal lobe surgery were employed as explanatory variables because patients with suspected temporal lobe epileptogenic zones had a lower chance of seizure freedom, as described before (R version 3.5.2, The R Foundation for Statistical Computing). A *p*-value of less than 0.05 was considered to be statistically significant.

### 2.10. Impact of Intracranial EEG on Surgical Strategy in the Patients with Focal MRI Lesion

We assessed whether the additional corticectomy beyond the extent of focal MRI lesion (extended lesionectomy) was performed based on the iEEG findings. The association between the extended cortical resection and seizure freedom was examined with Fisher’s exact test.

## 3. Results

### 3.1. Clinical Characteristics of Patients

The suspected epileptogenic zone was located within 1 or 2 lobes in 50 patients (94.3%), most frequently in the fronto-temporal region. The epileptogenic zone was lateralized without further indication of localization in three patients (5.7%). MRI revealed structural abnormalities in 45 patients (84.9%). Abnormal hypo- and hypermetabolism on FDG-PET was observed in 46 and 3 patients, respectively. The FDG-PET abnormalities were consistent with the MRI abnormalities in 39 patients (73.6%). The most common etiology was malformation of cortical development (64.2%), including 28 patients with focal cortical dysplasia (Table 1). The rationale for intracranial electrode implantation is summarized in Table 2. 

The majority of patients had MRI abnormalities in our study. Twenty-nine patients had a single MRI lesion with clear boundary (Focal MRI lesion). In our policy, iEEG was indicated even when the preoperative electro-clinical findings were not contradictory to the focal MRI lesion—in order to delineate the relationship between the seizure onset zone (SOZ) and the focal MRI lesion. It was hypothesized that better seizure outcome was achieved when the SOZ near or separate from the lesion were removed together. Sixteen patients had non-focal MRI lesions, including three cases with well-defined bilateral or multiple lesions, 10 cases with a diffuse lesion without clear boundary, and three cases with an ambiguous MRI finding, the significance of which radiologists were unsure of. Complete resection of the lesion was not expected, but partial resection of the epileptogenic zone was explored with iEEG in those cases. Eight patients (15.1%) had no MRI lesions. Five patients had apparently inconsistent electro-clinical findings to the lesion location. Functional cortical mapping was included in the purpose of iEEG in 15 cases. 

The number of electrodes and the method of implantation are summarized in Table 3.

### 3.2. Electrode Implantation

Depth electrodes were implanted using a neuronavigation system (VarioGuide^®^) in 48 patients (90.6%). The Leksell stereotactic frame was used in four patients including three with insulo-opercular lesion. Depth electrodes were manually inserted into the insular cortices after opening the Sylvian fissure in one patient. The median number of depth and subdural electrode leads was three and seven per patient, respectively. Severe complication of the increased intracranial pressure was observed in one patient (1.9%). The patient showed severe epidural hematoma and elevation of intracranial pressure at a day after electrode implantation and then needed evacuation of the hematoma and removal of implanted electrodes immediately. The epidural hematoma was not caused by insertion of depth electrodes. iEEG monitoring was terminated early in this patient before the electrodes were removed. This patient eventually underwent resective surgery four months after electrode removal and obtained seizure freedom without permanent neurological deficits. Depth electrodes were implanted into the deeply-located focal MRI lesions including bottom-of-sulcus type dysplasia (Figure 1, Figure 2 and Figure 3), into non-focal MRI lesion, or targeted to the non-lesional cortex where the symptomatogenic zone or the ictal propagation was suspected, or the non-lesional cortex with FDG-PET abnormalities as presented in Table 3. We postoperatively confirmed that the tips of depth electrodes were located in the gray matter of the target area.

### 3.3. Resective Surgery

All patients except one with severe complication underwent subsequent resective surgery at the time of electrode removal. Temporal lobe surgery was performed in all 15 patients with suspected epileptogenic zone in the temporal lobe, of whom four patients underwent anterior temporal lobectomy (ATL) with hippocampectomy, including one patient with extended resection of the lateral temporal cortex. Five patients underwent ATL without hippocampectomy to preserve the memory function. Five patients underwent focal cortical resection of the temporal neocortex, and one patient underwent selective resection of the uncus and amygdala (Table 1). Among the 38 patients with extra-temporal epileptogenic zone, 34 patients underwent focal cortical resection and/or lesionectomy, two patients underwent frontal lobectomy, one patient underwent parietal lobectomy, and one patient underwent occipital lobectomy.

### 3.4. Surgical Outcome

Seizure freedom (ILAE class 1) was achieved in 22 patients (41.5%), including three with suspected epileptogenic zone in the temporal lobe and 19 with extra-temporal epileptogenic zone (Table 4). 

Complete seizure freedom was significantly less common (*p* < 0.05, Fisher’s exact test) in the temporal lobe epilepsy (TLE) patients than in the extra-TLE patients. 

### 3.5. Detectability of EIIC

The EEG onset preceded the clinical onset in all patients. No significant difference in the detection of EIIC was found between subdural and depth electrodes (*p* = 0.23). Subdural electrodes detected EIIC in 45 patients (84.9%) and depth electrodes in 39 patients (73.6%) (Table 5). 

EIIC was observed in both subdural and depth electrodes in 31 patients (58.5%). EIIC was detected by only subdural electrodes in 14 patients (26.4%) and by only depth electrodes in eight patients (15.1%). EIIC was detected by only depth electrodes in all three patients who had depth electrodes in the insulo-opercular epileptogenic zone, and in two patients with TLE who had depth electrodes in the mesial temporal structures (Table 6). 

**Figure 4:**
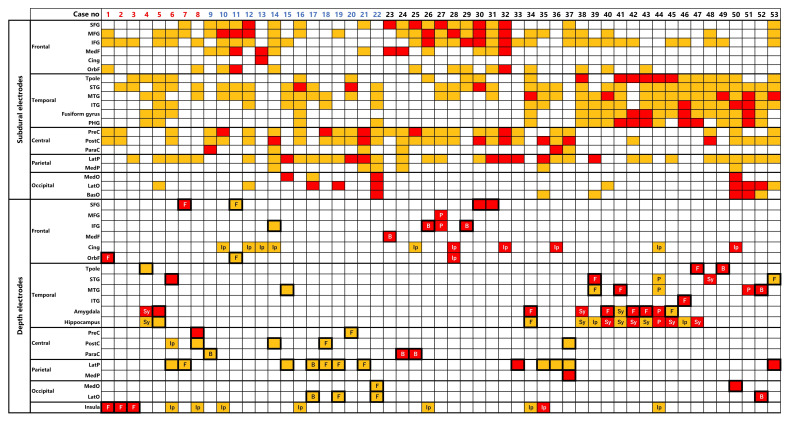
Anatomical locations of electrode are shown by color coding. The orange boxes refer to the area where the electrode was implanted but no EIIC was detected. The red boxes refer to the area where EIIC was detected. The bold squares indicate the region of MRI lesion. The cases of focal MRI lesion and of the bottom-of-sulcus FCD are denoted as “F” and “B”, respectively. The rationales of depth electrode implantation are denoted for non-lesional areas. EIIC was detected only by the depth electrodes in cases 1–8 and only by the subdural electrodes in cases 9–22. Abbreviations: B = bottom-of-sulcus type FCD, BasO = basal occipital cortex, Cing = cingulate gyrus, F = focal MRI lesion, IFG = inferior temporal gyrus, Ip = ictal propagation, ITG = inferior temporal gyrus, LatO = lateral occipital cortex, LatP = lateral parietal cortex, MedF = medial frontal cortex, MedO = medial occipital cortex, MedP = medial parietal cortex, MFG = middle frontal gyrus, MTG = middle temporal gyrus, OrbF = orbitofrontal cortex, P = PET-positive lesion, ParaC = paracentral lobule, PHG = parahippocampal gyrus, PreC = precentral gyrus, PostC = postcentral gyrus, SFG = superior frontal gyrus, STG = superior temporal gyrus, Sy=symptomatogenic cortex, Tpole = temporal pole.

Anatomical locations of electrodes and EIIC findings are summarized in Figure 4.

EIIC was localized in sub-lobar level but was observed both in subdural and depth electrodes in the majority of the cases. Subdural electrodes detected EIIC in 25 of the 29 patients with a focal MRI lesion and did so in all 10 patients with bottom-of-sulcus FCD, although the lesion was simultaneously explored by depth electrodes. No clear superiority of the combined depth electrode in detecting EIIC was demonstrated.

### 3.6. Morphological Patterns of EIIC

The four types of morphological pattern of EIIC were observed and showed no clear difference between the subdural and depth electrodes, except that rhythmic delta activity was only detected by the subdural electrodes (Table 7). 

LVFA was the most common pattern seen in 41 patients (77.4%). Subdural electrodes detected LVFA in 35 patients, and depth electrodes in 33 patients (*p* = 0.58). Thirteen of the 41 patients demonstrated the “start-stop-start phenomenon”, which was characterized by leading spikes (or polyspikes) with subsequent attenuation antecedent to the LVFA [9]. Other morphological patterns were observed as follows: rhythmic delta activity in seven patients (13.2%), spike-and-wave complex in four (7.5%), and rhythmic theta activity with preceding polyspikes in one (1.9%).

### 3.7. Factors Associated with Detectability of EIIC and LVFA

#### 3.7.1. Etiology

The etiology was associated with the detectability of EIIC in depth electrodes. EIIC was detected with depth electrodes less often in patients with ulegyria than with other etiologies (*p* < 0.005, Fisher’s exact test). Depth electrodes failed to detect EIIC in five of six patients with ulegyria, although the depth electrodes were inserted into the ulegyria lesion in all patients. Depth electrodes detected EIIC in 26 of 34 patients with malformation of cortical development (76.5%) and in 12 of 13 patients with other etiologies (92.3%). Subdural electrodes detected EIIC in 28 of 34 patients (82.4%) with malformation of cortical development, in all six patients with ulegyria, and in 11 of 13 patients (84.6%) with other etiologies (Table 5). The detectability of EIIC in subdural electrodes was not associated with the etiology.

#### 3.7.2. Suspected Temporal Lobe Epileptogenic Zone

The detectability of EIIC and LVFA was not different between TLE and extra-TLE (Table 5), although complete seizure freedom was significantly less common in TLE patients than in extra-TLE patients (Table 4).

#### 3.7.3. Other Factors

Detectability of EIIC and LVFA was not associated with other factors, including single lobe MRI lesion and the concordance of FDG-PET abnormality with MRI lesion (Table 5).

### 3.8. Association between the Detectability of EIIC and Seizure Freedom

The detectability of EIIC was not associated with the achievement of seizure freedom. Seizure freedom (ILAE class 1) was obtained in 44.4% of patients in whom subdural electrodes detected EIIC and 35.9% of patients in whom depth electrodes detected EIIC. The seizure-free rate was 41.5% in patients with LVFA and 38.5% in patients with leading spikes. Detection of LVFA and leading spikes was not associated with seizure freedom.

### 3.9. Factors Related to the Chance of Seizure Freedom

Multiple logistic regression analysis revealed that postoperative seizure outcome was predictable by the complete removal of MRI lesion (*p* < 0.01) and temporal lobe epileptogenic zone (*p* < 0.05) but not by the complete removal of EIIC (*p* = 0.56) (Table 8).

The MRI lesion was totally resected in 28 patients (52.8%), including 24 patients with focal MRI lesion and four patients with non-focal MRI lesion. The seizure-free rate was 57.1% in those 28 patients and was 24.0% in the remaining patients. The EIIC was completely resected in 45 patients (84.9%). Partial resection of EIIC was indicated in eight patients to preserve the functional regions including the hippocampus and the primary sensorimotor cortices. Three of them showed diffusely distributed EIIC. The seizure-free rate was 42.2% in the patients whose EIIC was completely removed. Three patients achieved seizure freedom after partial resection of EIIC (37.5%).

## 4. Discussion

Addition of depth electrodes to subdural electrodes in combination was less useful than we had expected in terms of detection of EIIC. Depth electrodes were useful for confirming the seizure onset from the suspected deep lesion. However, additional depth electrodes were superior to subdural electrodes only in certain cases, especially patients with insulo-opercular lesion. Subdural electrodes were superior to depth electrodes in patients with ulegyria. The significance of the removal of EIIC is only marginal compared with the removal of MRI lesion. The poor outcome in TLE patients in this study raises questions about the significance of our strategy for the patients with suspected temporal lobe epileptogenic zone. 

### 4.1. Significance of Adding Depth Electrodes to Subdural Electrode Implantation in Detection of EIIC

No clear superiority for subdural or depth electrodes for the detection of EIIC and LVFA was observed in this study. The significance of implantation of depth electrodes additional to subdural electrodes was limited and only confirmed that the combined use of subdural and depth electrodes is sensitive for detecting EIIC. One reason is that exploration was limited by the extent of craniotomy. Consequently, EIIC tends to appear almost simultaneously in both depth and subdural electrodes because the two electrodes are located in relatively close proximity. This limitation does not necessarily negate the usefulness of the additional depth electrode, which provides spatially dense monitoring of the suspected epileptogenic area. However, a method is needed to determine which of the findings that appear on both depth and subdural electrodes is more important to identify the epileptogenic zone and to plan the resective surgery.

EIIC was detected by only depth electrodes in eight patients, including all three patients with insulo-opercular epileptogenic zone and two patients with TLE. Therefore, the additional depth electrode is useful for detecting EIIC from apparently deep structures such as the insula and mesial temporal structures.

### 4.2. Etiology Unsuitable for Additional Depth Electrodes

Depth and subdural electrodes may be used differently depending on the etiology. EIIC was less well detected with depth electrodes in patients with ulegyria than with the other etiologies. This finding implies that epileptic activities are not generated within the ulegyria lesion but in the surrounding cortex. Neuronal cells are depleted within the ulegyria lesion and so may not be able to function as a seizure onset zone [10]. Therefore, applying subdural electrodes around the ulegyria lesion may be useful to detect EIIC and observe ictal propagation, which is helpful for planning selective resection, especially if cortical function should be preserved as much as possible [11]. Depth electrodes should be carefully used after consideration of the dependence of efficacy on the etiology to avoid injudicious usage. Further study is required for other etiologies because the small number of cases did not allow for statistical investigation.

### 4.3. Association with Seizure Outcome

Our series showed no association between the detection of EIIC and seizure outcome. From the results of multivariate analysis, the complete resection of MRI lesion was the most contributing factor for seizure freedom, but the complete resection of EIIC was not. However, it is very difficult to conclude that complete resection of EIIC was meaningless from this result. We believe that the low statistical power and the confounding factor made it difficult to prove the significance of EIIC removal. Complete resection of MRI lesions was achieved in only 52.8% of patients. In contrast, complete resection of EIIC was performed in most patients (84.9%), leaving not enough comparators to establish statistical significance. EIIC was only observed at the location where electrodes were placed and was always removed except in the case of overlapping functional area. It is also very likely that the true EIIC could be missed when it occurred outside the electrode coverage and was not included in the resection, which might influence the outcome. The seizure outcome is not only influenced by the detection of EIIC, but largely by where and how electrodes are implanted.

The detection of LVFA was also not associated with seizure outcome, although LVFA is the most common morphological pattern of EIIC and previous studies reported that the detection of LVFA was associated with seizure outcomes [12,13,14].

Relatively low seizure-free rate in our study is partly explained by inclusion of patients with non-focal MRI lesion. We included 16 patients (30.2%) with bilateral, multifocal, and diffuse MRI lesion or ambiguous MRI findings. Complete removal of MRI lesion was achieved only in 28 patients (52.8%). 

### 4.4. Exploration of Suspected TLE

In our study, patients with suspected TLE who required electrode implantation had poorer seizure outcome than patients with extra-TLE. This result contradicts the experience that surgical treatment for TLE generally provides good postoperative outcomes [15,16,17,18,19,20]. It is likely that our combined depth and subdural approach often failed to identify the epileptogenic zone in patients with suspected TLE. Some patients possibly had temporal “plus” epilepsy, which is associated with unfavorable seizure outcome after temporal lobe surgery [21]. Therefore, broader exploration that is not limited to the temporal lobe may be recommended in patients with suspected TLE. SEEG is reported to contribute to favorable seizure control even in temporal “plus” epilepsy [22,23]. Resective surgery is not recommended in patients who suffer independent seizures from the bilateral temporal regions identified on scalp EEG but with inconclusive findings from all other evaluations [24]. Careful indication for our approach is necessary in patients with suspected TLE.

### 4.5. Advantages and Limitations of Combined Electrode Implantation 

Combined implantation of subdural and depth electrodes provides detailed and dense information about a relatively small area of the brain. This method is suitable for more precise determination of the extent of resection [25,26]. In our series, iEEG altered the surgical strategy in six of the 29 patients with focal MRI lesion. Extended lesionectomy based on the iEEG findings may maximize the chances of seizure freedom, although iEEG did not change the policy of lesionectomy in the majority of cases. Further study is necessary to prove the value of our approach in patients with MRI lesion. 

The combination of both subdural and depth electrodes has been reported in several literatures [27,28,29,30], including a series with additional depth electrodes for the exploration of hippocampal activity [29,30,31]. Since the 1990s, it has been reported that depth electrodes implanted in the hippocampus detected ictal activity earlier than subdural electrodes placed on the lateral temporal cortex [29,30]. Some reports insisted that the combined electrode implantation can provide better understanding of the origin and propagation of ictal activity than either method alone in extra-temporal lobe epilepsy [25,32]. In our study, additional depth electrodes were only useful to explore the earlier ictal activity occurring in the mesial temporal structures or in the insula. However, the usefulness was unclear for the other neocortical regions or for the depth-of-sulcus lesions. 

Few series have described the postoperative seizure outcomes in the patients with combined electrode implantation. In our series, 41.5% of the patients achieved seizure freedom (ILAE class 1), and 67.9% had favorable seizure outcome (ILAE classes 1–3). Nagahama et al. reported the seizure-free (Engel class 1) rate of 65.3% in a series with combined subdural and depth electrode implantation [32]. The series contained many TLE cases, including patients diagnosed with mesial temporal sclerosis (22.0%), and 72.2% of all patients ultimately underwent ATL or extended ATL. In addition, 65.3% of patients underwent lesionectomy, with a higher proportion of patients undergoing complete lesionectomy than in our series. Bulacio et al. reported the seizure-free (ILAE class 1) rate was 61% at one year after resective surgery, but decreased to 47% at three years, 42% at five years, and 33% at 10 years [33]. 

### 4.6. Limitation of Study

This study contains several limitations. The first is its retrospective nature. Indication, planning, and techniques of electrode implantation vary from institution to institution. Therefore, it is necessary to consider institutional bias and the difficulty of generalizing our results. Second, the number of patients was limited; thus, statistical evaluation could not be sufficiently performed between cerebral regions and between etiologies. 

Third, we failed to uncover the relationship between the detection of EIIC by depth or subdural electrodes and seizure outcome. As discussed in Section 4.3, it is inherently difficult to bring the control necessary for the resection of EIIC. Seizure outcomes are largely influenced by where and how electrodes are implanted, beyond the detection of EIIC. We found in this study that seizure outcome was paradoxically poor in patients with suspected temporal lobe epilepsy, suggesting our approach is not appropriate in those patients. This result does not negate the usefulness of additional depth electrodes to the mesial temporal region but challenges the usefulness of our approach. 

Fourth, EIIC may not be appropriate to prove the overall usefulness of depth or subdural electrodes. There are no established methods for defining the area of EEG seizure onset, and the EIIC was visually assessed in this study. The usefulness of depth electrodes could be found by using different but objective biomarkers such as high-frequency oscillations or DC shift. In a previous article to which we referred in defining EIIC [7], the authors did not investigate the association between EIIC and surgical outcomes. 

## 5. Conclusions

The addition of depth electrodes to subdural electrodes has limited value for detection of EIIC. Additional depth electrodes provide spatially dense monitoring of the suspected epileptogenic area. Depth electrodes should be considered for the exploration of apparently deep locations, such as the insula and mesial temporal structures. On the other hand, they are less effective in the gyrus or ulegyria covered by subdural electrodes and should be used with caution.

## Figures and Tables

**Table 1:** Clinical characteristics.

**Age, mean (range), years**	**17.6 (3–53)**
**Female sex, n (%)**	28 (52.8)
**Follow-up duration after resective surgery, mean (range), months**	27.5 (12.2–49.5)
**Seizure semiology, *n* (%)**	
Impaired awareness or behavior arrest	24 (45.3)
Tonic	19 (35.8)
Hyperkinetic	8 (15.1)
Versive	7 (13.2)
Clonic	4 (7.5)
Epileptic spasms	4 (7.5)
Auras	18 (34.0)
Focal to bilateral tonic-clonic	5 (9.4)
**Side of estimated epileptogenic zone, *n* (%)**	
Right	29 (54.7)
Left	23 (43.4)
Undetermined	1 (1.9)
**Localization of estimated epileptogenic zone, *n* (%)**	
Localized in 1–2 lobes	50 (94.3)
Frontal	14 (26.4)
Temporal	15 (28.3)
Fronto-temporal	1 (1.9)
Fronto-parietal	5 (9.4)
Parietal	6 (11.3)
Temporo-occipital	4 (7.5)
Insulo-opercular	3 (5.7)
Occipital	1 (1.9)
Temporo-parietal	1 (1.9)
Lateralized in unilateral hemisphere	3 (5.7)
**FDG-PET abnormality, *n* (%)**	49 (92.5)
Consistent localization with MRI lesion	39 (73.6)
Inconsistent localization with MRI lesion	6 (11.3)
No concomitant MRI lesion	4 (7.5)
**Resective surgery, *n* (%)**	
Temporal lobe surgery	
ATL with hippocampectomy	4 (7.5)
ATL without hippocampectomy	5 (9.4)
Focal cortical resection and/or lesionectomy	5 (9.4)
Selective resection of uncus and amygdala	1 (1.9)
Extra-temporal lobe surgery	
Focal cortical resection and/or lesionectomy	34 (64.2)
Lobectomy	4 (7.5)
**Etiology of epilepsy, *n* (%)**	
MCD	34 (64.2)
Focal cortical dysplasia	28 (52.8)
Type 1	6 (11.3)
Type 2	18 (34.0)
Microdysgenesis	4 (7.5)
Polymicrogyria	1 (1.9)
Tuberous sclerosis	1 (1.9)
Other MCD	4 (7.5)
Ulegyria	6 (11.3)
Hippocampal sclerosis	2 (3.8)
Middle fossa encephalocele	1 (1.9)
Arteriovenous malformation	1 (1.9)
Tumor	1 (1.9)
Unknown	8 (15.1)

ATL, anterior temporal lobectomy; FDG-PET, ^18^F-fluorodeoxyglucose-positron emission tomography; MCD, malformation of cortical development; MRI, magnetic resonance imaging; TLE, temporal lobe epilepsy.

**Table 2:** Rationale for electrode implantation stratified by MRI findings.

**1. Focal MRI lesion**	**29 (54.7)**
Inconsistent electro-clinical findings	2 (3.8)
Functional mapping	14 (26.4)
**2. Non-focal MRI lesion**	**16 (30.2)**
**Bilateral MRI lesions**	**2 (3.8)**
Inconsistent electro-clinical findings	1 (1.9)
**Multiple MRI lesions**	**1 (1.9)**
**Diffuse MRI lesion**	**10 (18.9)**
Inconsistent electro-clinical findings	1 (1.9)
Functional mapping	1 (1.9)
**Ambiguous MRI findings**	**3 (5.7)**
Inconsistent electro-clinical findings	1 (1.9)
**3. No MRI lesions**	**8 (15.1)**

MRI, magnetic resonance imaging.

**Table 3:** Details of electrode implantation.

**No. of Depth Electrode Implantation, Median (Range)**	
Depth electrode leads per patient	3 (1–10)
Depth electrode contacts per patient	22 (6–60)
**Method of depth electrode implantation, n (%)**	
VarioGuide^®^	48 (90.6)
Leksell stereotactic frame	4 (7.5)
Manual insertion	1 (1.9)
**Target of depth electrode, number of leads (number of patients)**	
Focal MRI lesion	83 (29)
Bottom-of-sulcus FCD	33 (10)
Non-focal MRI lesion	39 (14)
Non-lesional cortex	71 (30)
Symptomatogenic cortex	14 (9)
Suspected area of ictal propagation	47 (19)
Cortex with FDG-PET abnormality	10 (3)
**No. of subdural electrode implantation, median (range)**	
Subdural electrode leads per patient	7 (2–16)
Subdural electrode contacts per patient	62 (10–136)

FDG-PET, ^18^F-fluorodeoxyglucose-positron emission tomography; MRI, magnetic resonance imaging; FCD, focal cortical dysplasia.

**Table 4:** Postoperative seizure outcome.

ILAE Class	Overall (*n* = 53) *n* (%)	TLE (*n* = 15) *n* (%)	Extra-TLE (*n* = 38) *n* (%)
**1**	**22 (41.5)**	3 (20.0) *	19 (50.0) *
1a	20 (37.7)	2 (13.3)	18 (47.4)
2	6 (11.3)	3 (20.0)	3 (7.9)
3	8 (15.1)	1 (6.7)	7 (18.4)
4	12 (22.6)	6 (40.0)	6 (15.8)
5	4 (7.5)	2 (13.3)	2 (5.3)
6	1 (1.9)	0	1 (2.6)

* *p* < 0.05 (Fisher’s exact test). ILAE, International League Against Epilepsy; TLE, temporal lobe epilepsy.

**Table 5:** Detectability of EIIC and LVFA.

	EIIC Detectability, *n* (%)		LVFA Detectability, *n* (%)
	Depth Electrodes	Subdural Electrodes	
Overall	39 (73.6)	45 (84.9)	41 (77.4)
Etiology			
Malformation of cortical development (*n* = 34)	26 (76.5)	28 (82.4)	26 (76.5)
Ulegyria (*n* = 6)	1 (16.7) *	6 (100)	4 (66.7)
Other etiologies (*n* = 13)	12 (92.3)	11 (84.6)	11 (84.6)
Location of suspected epileptogenic zone			
TLE (*n* = 15)	15 (100)	12 (80.0)	14 (93.3)
Extra-TLE (*n* = 38)	24 (63.2)	33 (86.8)	27 (71.1)
MRI lesion localized in a single lobe (*n* = 31)	23 (74.2)	28 (90.3)	28 (90.3)
FDG-PET findings consistent with MRI (*n* = 39)	28 (71.8)	33 (84.6)	32 (82.1)

* *p* < 0.05 (Fisher’s exact test). EIIC, early ictal intracranial electroencephalographic change; FDG-PET, ^18^F-fluorodeoxyglucose-position emission tomography; LVFA, low-voltage fast activity; MRI, magnetic resonance imaging; TLE, temporal lobe epilepsy.

**Table 6:** Patients with EIIC detected by only depth electrodes.

Case No.	Age-Range, Years	Etiology	Estimated Epileptogenic Zone	MRI Lesion	FDG-PET Lesion	Target of Subdural Electrode	Target of Depth Electrodes (No. of Leads Showing EIIC/No. of Leads)	Method of Depth Electrode Implantation	Seizure Outcome (ILAE)
1	20–25	FCD	L IO	L IO	None	L FP	L insula (5/5) Orbitofrontal (1/1)	LSF	4
2	0–5	FCD	L IO	L IO	L IO	L FTP	L insula (1/4)	LSF	1a
3	6–10	FCD	R IO	R insula	R insula	R FT	R insula (2/4)	LSF	1a
4	20–25	Other MCD	R T	R T/L F	R T	R FT	R hippo (0/1) R amygdala (1/1) MRI lesion (R T) (0/1)	VarioGuide	4
5	40–45	Unknown	R T	R T	R T	Bil FTP	Bil hippo (1/2) Bil amygdala (0/2)	VarioGuide	2
6	50–55	AVM	R T	R T	R T	R FTP	MRI lesion (R T) (1/3) R cingulate (0/2) R insula (0/1)	VarioGuide	4
7	0–5	FCD	R FP	R FP	R FP	R FP	MRI lesion (R FP) (1/4)	VarioGuide	3
8	10–15	FCD	L FP	L hemi	L FTP	L FP	MRI lesion (L FP) (1/2) L insula (0/5)	VarioGuide	1a

AVM, arteriovenous malformation; Bil, bilateral; EIIC, early ictal intracranial electroencephalographic change; F, frontal; FCD, focal cortical dysplasia; FDG-PET, ^18^F-fluorodeoxyglucose-position emission tomography; FP, fronto-parietal; FTP, fronto-temporo-parietal; hemi, hemisphere; hippo, hippocampus; ILAE, International League Association of Epilepsy; IO, insulo-opercular; L, left; LSF, Leksell stereotactic frame; MCD, malformation of cortical development; MRI, magnetic resonance imaging; R, right; T, temporal.

**Table 7:** Morphological patterns of EIIC.

	Subdural Electrodes, *n* (%)	Depth Electrodes, *n* (%)	*p*-Value
LVFA (*n* = 41)	35 (77.8)	33 (84.6)	0.58
Rhythmic delta activity (*n* = 7)	7 (15.6)	1 (2.6)	0.062
Spike-and-wave complex (*n* = 4)	3 (6.7)	4 (10.3)	0.70
Rhythmic theta activity with preceding polyspikes (*n* = 1)	0	1 (2.6)	0.46

EIIC, early ictal intracranial electroencephalographic change; LVFA, low-voltage fast activity.

**Table 8:** Multiple logistic regression analysis for seizure freedom.

Variable	Odds Ratio	95% CI	*p*-Value
(Intercept)	0.61	0.127 to 2.93	0.54
Complete removal of MRI lesion	6.32	1.62 to 24.7	0.0079
Complete resection of EIIC	0.61	0.114 to 3.26	0.56
Suspected temporal lobe epileptogenic zone	0.15	0.0297 to 0.775	0.023

EIIC, early intracranial electroencephalographic change; MRI, magnetic resonance imaging.

## Data Availability

Requests for data will be considered upon request.
